# Parental thermal environment controls the offspring phenotype in Brook charr (*Salvelinus fontinalis*): insights from a transcriptomic study

**DOI:** 10.1093/g3journal/jkae051

**Published:** 2024-03-13

**Authors:** Ghizlane Banousse, Eric Normandeau, Christina Semeniuk, Louis Bernatchez, Céline Audet

**Affiliations:** Institut des sciences de la mer de Rimouski (ISMER), Université du Québec à Rimouski (UQAR), Rimouski, QC, Canada G5L 2Z9; Plateforme de bio-informatique de l’IBIS (Institut de Biologie Intégrative et des Systèmes), Université Laval, Québec, QC, Canada G1V 0A6; Great Lakes Institute for Environmental Research (GLIER), University of Windsor, Windsor, Ont, Canada N9C 1A2; Institut de Biologie Intégrative et des Systèmes (IBIS), Université Laval, Québec, QC, G1V 0A6, Canada; Institut des sciences de la mer de Rimouski (ISMER), Université du Québec à Rimouski (UQAR), Rimouski, QC, Canada G5L 2Z9

**Keywords:** Brook charr, epigenetics, brain, fry, temperature

## Abstract

Brook charr is a cold-water species which is highly sensitive to increased water temperatures, such as those associated with climate change. Environmental variation can potentially induce phenotypic changes that are inherited across generations, for instance, via epigenetic mechanisms. Here, we tested whether parental thermal regimes (intergenerational plasticity) and offspring-rearing temperatures (within-generational plasticity) modify the brain transcriptome of Brook charr progeny (fry stage). Parents were exposed to either cold or warm temperatures during final gonad maturation and their progeny were reared at 5 or 8 °C during the first stages of development. Illumina Novaseq6000 was used to sequence the brain transcriptome at the yolk sac resorption stage. The number of differentially expressed genes was very low when comparing fry reared at different temperatures (79 differentially expressed genes). In contrast, 9,050 differentially expressed genes were significantly differentially expressed between fry issued from parents exposed to either cold or warm temperatures. There was a significant downregulation of processes related to neural and synaptic activity in fry originating from the warm parental group vs fry from the cold parental one. We also observed significant upregulation of DNA methylation genes and of the most salient processes associated with compensation to warming, such as metabolism, cellular response to stress, and adaptive immunity.

## Introduction

The global warming rate is now higher than ever. In Canada, the global surface temperature has increased by 1.07 °C since the onset of the industrial period, and a further increase of 1.4 °C is predicted by 2100 (IPCC 2022). Temperature is the environmental factor that affects the most physiology and behavior in ectotherms, including fishes (Fry 1947; Woodin *et al*. 2013; Teal *et al*. 2018; Christensen *et al*. 2021). Moreover, several studies have shown that elevated water temperatures may have detrimental effects on fish metabolism (Pörtner 2001), fecundity and reproduction (Lahnsteiner and Leitner 2013; Fenkes *et al*. 2016), growth (Prokešová *et al*. 2020), behavior, and neurobiology (Biro *et al.* 2007; Babkiewicz *et al*. 2021). As such, there is a pressing need to evaluate the different species-specific adaptive responses and predict their persistence and resilience to climate change (Crozier and Hutchings 2014).

One way that organisms can cope with environmental fluctuations is through phenotypic plasticity, a fast-acting mechanism that refers to the ability of an organism to alter its phenotype, including physiological, behavioral, and morphological traits, in response to changes in environmental conditions (Thompson 1991; Pigliucci 2005; West-Eberhard 2003). Phenotypic plasticity has long been recognized as being ubiquitous in aquatic species (Grabowski *et al.* 2009; Koumoundouros *et al*. 2009; Potts *et al*. 2021), particularly in salmonids (Hutchings 1996; Karjalainen *et al.* 2016); and numerous examples of thermal plasticity during different life stages have been documented (Solberg *et al*. 2016; Hvas *et al*. 2017). In previous studies, it has been recognized that within-generational plasticity is likely to play an essential role in allowing organisms to compensate for climate change effects and that adaptive plasticity during early ontogeny (developmental plasticity) is most critical (Gilbert 2001; West-Eberhard 2003; Bateson *et al.* 2014). Nevertheless, as global warming trends will span multiple generations, emerging studies are focusing on exploring the adaptive potential of multigenerational plasticity, where parental environment shapes offspring physiological and behavioral phenotypes (Toyota et al. 2019; Anastasiadi *et al.* 2021). Observations of multigenerational plasticity have been documented in various organisms (Donelson *et al.* 2018) and suggest that some detrimental effects of elevated temperature can be compensated for when warming is experienced across multiple generations, allowing genetic adaptation to occur over the longer term (Salinas and Munch 2012; Torda *et al.* 2017). However, while most of our knowledge is based on observations at the phenotypic level, the underlying molecular signatures of plasticity within and across generations remain unclear.

Transcriptional modulation through epigenetics is likely to be the primary molecular mechanism underlying phenotypic plasticity (Leitwein *et al.* 2021). Modifications in organisms’ environmental conditions can cause epigenetic regulation such as DNA methylation (Lister *et al.* 2009), histone modifications (Bannister and Kouzarides 2011), small interfering RNA molecules (Costa 2008), and modification of locus topography in the nucleus (Cremer and Cremer 2010). Such regulation may in turn affect gene expression, potentially contributing to the ability of organisms to exhibit phenotypic plasticity in response to environmental cues (Leitwein *et al.* 2021). However, given the periods of large-scale epigenomic reprogramming (epigenetic/transcriptomic patterns derived from the parental germ cells are erased and replaced with new markers specific to the embryo), the major unresolved question remains whether these epigenetic and transcriptomic changes can persist and be transmitted across generations. In mammals, there is evidence that some parts of the genome may resist global epigenomic reprogramming. This may provide a putative route for epigenetic states and therefore, gene expression patterns to be transmitted from one generation to the next (Tuscher and Day 2019). However, in teleost fishes, epigenomic reprogramming is still not as well-established as in mammals and differs among species. For example, while in zebrafish (*Danio rerio*) epigenetic information (e.g. DNA methylation patterns) does not appear to exhibit a reprogramming (Jiang *et al.* 2013), evidence from medaka (*Oryzias latipes*) showed a similar reprogramming of epigenetic information to what is observed in mammals (Wang and Bhandari 2019). This lack of consistency between species makes teleosts an interesting model to further investigate the potential of multigenerational plasticity. To date, few examples have studied the effect of elevated temperatures at the transcriptional level (Shama *et al*. 2016; Bernal *et al*. 2018; Bernal, Ravasi, *et al.* 2022; Veilleux *et al*. 2018). For example, Penney (2024) found that multigenerational thermal plasticity alone or combined with within-generational thermal plasticity in Lake trout (*Salvelinus namaycush*), can influence the differentially expressed genes (DEGs) associated with some compensatory processes to warming, such as metabolism and thermal stress tolerance.

Most of the studies evaluating transcriptional thermal plasticity, either within or across generations, focus on the liver, muscle, and gonads (Shama *et al.* 2016; Bernal *et al*. 2018; Bernal, Ravasi, *et al.* 2022; Penney 2024; Veilleux *et al*. 2018). In the context of the thermal response, the brain tissue, however, should play a central role in orchestrating several physiological (metabolism, stress response, immune function) (Crawshaw *et al*. 1985), neurobiological (O’Donnell 2018), and thermoregulatory behavioral responses (Volkoff and Rønnestad 2020). Only a few studies have investigated the molecular response of the brain to temperature variations using an RNA sequencing approach, such as in the Spiny chromis (*Acanthochromis polyacanthus*) (Bernal, Schmidt, *et al*. 2022), and in Red seabream (*Pagrus major*) (Okuzawa and Gen 2013), and showed an alteration of the brain gene expression patterns in response to water temperature variations. These altered gene expressions were associated with processes related to neuroplasticity, endocrine signaling, metabolism, immunity, and cellular stress response. Given the central and diverse role of the brain in response to environmental variation, exploring its molecular response to projected temperatures could yield new insights into the thermal response both within and across generations.

Brook charr (*Salvelinus fontinalis*) is a cold-adapted salmonid native to eastern North America that is largely limited to cold (<20°C) and well-oxygenated lakes and streams (Scott and Crossman 1973). This species is highly sensitive to increased water temperatures (Cook *et al*. 2018) and climate change is already impacting much of the Brook charr populations (Stanfield *et al*. 2006). Brook charr is also an economically important species in Eastern Canada. It supports recreational fishing and represents approximately 70% of fish production intended for stocking in Québec (MFFP 2019). Thus, considering its vulnerability to global warming and its economic importance, it is a relevant species in which to explore the generality of its molecular response within and across generations to projected warming conditions.

Previous studies that have explored thermal plasticity at the molecular level in Brook charr showed an ability to acclimate to high temperatures with the restoration of the aerobic register, and an alteration in the expression of specific genes involved in cellular stress response (Stitt *et al.* 2014; Mackey *et al*. 2021), osmoregulation, and oxidative stress (Mackey 2021), along with the presence of intraspecific variations between populations (Stitt *et al.* 2014). However, most of these studies focused on within-generational thermal plasticity, and multigenerational plasticity remains largely unexplored. Using the same experimental design as the one we used in this study, Venney *et al.* (2022) investigated the intergenerational epigenetic response of Brook charr to elevated temperatures. Their results revealed a strong influence of parental temperature during gonad maturation on liver DNA methylation in offspring at the yolk sac resorption stage, which suggested an intergenerational epigenetic inheritance. Considering the central and diverse role of the brain in initiating a range of neurobiological, physiological, and behavioral responses to environmental changes, we aimed to study how intergenerational epigenetics through transcriptional change would be expressed in the brain of Brook charr fry sampled at the same stage of development.

In this study, we sampled brain tissue from Brook charr fry reared under two thermal regimes, 5 and 8 °C, and whose parents were exposed to one of two thermal gradient regimes during gonadal maturation: cold and warm. Using RNA sequencing, we sought to evaluate the effects of parental temperature relative to offspring temperature and therefore investigate the molecular basis of within and inter-generational response of Brook charr to elevated temperatures. Based on the findings of Venney *et al.* (2022), we hypothesized that parental temperature and not offspring-rearing temperature would induce modifications in the expression patterns of neural genes related to DNA methylation, metabolism, immunity, cellular stress response, and neuroplasticity.

## Materials and methods

### Animals and experimental design

**Fig. 1.**
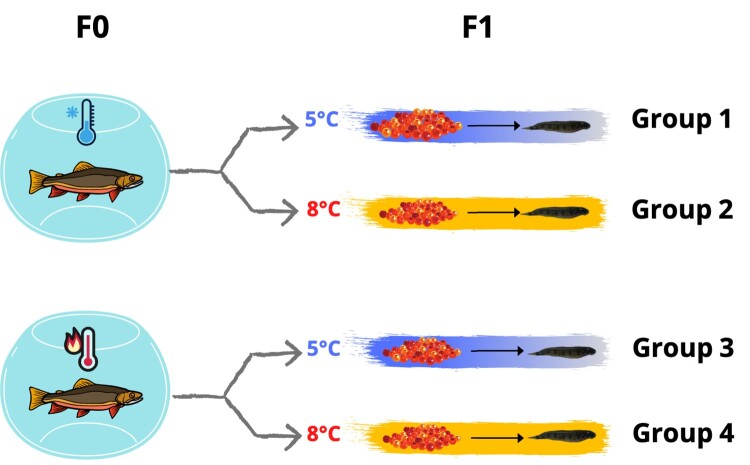
Experimental design for thermal treatments in Brook charr. Brook charr adults (F0) were exposed to two different thermal regimes separated by 2 °C during the final stages of gonadal maturation: Cold (top) and warm (bottom). Fertilized eggs (F1) from each parental thermal group were split into two batches; one was incubated at 5 °C and the second one was incubated at 8 °C until yolk sac resorption.

Breeders used in this experiment came the Brook charr *Laval* strain that was kept captive for five generations at the *Station aquicole de Pointe-au-Père* (ISMER–UQAR, Québec, Canada; 48°27N, 68°32W). This strain originated from a wild anadromous population established in the Laval River on the north shore of the St. Lawrence estuary, Québec (48°44N, 69°05W). During gonadal maturation, the adult Brook charr held at the *Station aquicole* ISMER–UQAR were split between two parental groups and were exposed to two different thermal regimes separated by 2 °C: cold (from 11.5 °C in September to 3 °C in December) and warm (from 13.5 °C in September to 5 °C in December). At the time of spawning, and for each of the parental groups, five “2 × 2” semifactorial crosses were carried out: the eggs of each female were separated into two batches, each being fertilized by a different male and the two males also being used to fertilize the eggs of a second female. Sixteen families were produced from the cold-parental group, and 21 from the warm-parental group, the unbalanced family number in each group being explained by the variable number of sexually mature males and females at each spawning event. The fertilized eggs of the two parental groups were then transferred to the LARSEM facilities at Laval University (Québec City, QC, Canada). Eggs from each family of the two parental groups were split into two batches, one being incubated at 5 °C and the second one at 8 °C, each family being kept separately. The same thermal conditions were maintained from hatching to yolk sac resorption. Thermal conditions were based on natural freshwater regimes in our area and on previous experiments we conducted in our facilities. For the parental groups, a temperature difference of 2 °C was maintained to reflect the expected freshwater warming expected in Canada by 2100 under a low-emission scenario (RCP 2.6; Zhang *et al*. 2019). For the offspring, 5 °C was chosen for incubation simply because it is the intermediate temperature we previously used in production during this period of development, while 8 °C is the temperature condition we previously used at first feeding (Bastien *et al*. 2011; Crespel *et al*. 2011; Granier *et al*. 2011). We then worked with a temperature interval in which we knew based on previous experience we would have thermal conditions ensuring normal early development and the absence of fungus infections or surge of bacterial opportunistic infections. At the exogenous feeding stage, 10 fry from each family were sampled and stored at −80 °C for future analysis. This experimental design resulted in four experimental groups: group 1 (5 °C reared offspring with cold-acclimated parents), group 2 (8 °C reared offspring with cold-acclimated parents), group 3 (5 °C reared offspring with warm-acclimated parents), and group 4 (8 °C reared offspring with warm-acclimated parents) (Fig. 1). In the current study, 67 fry representing the different experimental groups were randomly selected: 14 samples from group 1, 16 samples from group 2, 19 samples from group 3, and 18 samples from group 4. Only samples with sufficient RNA concentration for sequencing were selected which resulted in an unbalanced number of samples among the groups.

### RNA extraction, library preparation, and RNA sequencing

Fry brain tissues were dissected out on ice under a microscope (Leica MZ95). Total RNA was then extracted with the RNeasy Fibrous Tissue Kit (Qiagen. Inc., Mississauga, ON, Canada) following manufacturer instructions. RNA concentration and purity were verified (260/280 ratio was between 2.07 and 2.09) using a Nanodrop ND-1000 Spectrophotometer version 3.3.0 (NanoDrop Technologies, Inc., Delaware, USA). RNA quality was assessed by SYBR Safe DNA Gel Stain 2% agarose gel electrophoresis (Alpha Imager HP System, Alpha-Innotech, Alpha Software, Invitrogen, Inc., CA, USA). Then RNA samples were stored at −80 °C before sequencing. The total RNA extracted was normalized to ∼50–100 ng μL^−1^ at a total volume of ∼24–144 μL and sent to the *Centre d’expertise et de services Génome Québec*, Montréal, Canada. Confirmatory quality control of RNA samples was carried out using a Bioanalyser, and for all samples, the RNA integrity number (RIN) was higher than 7. RNA-Seq libraries for paired-end fragments of 100 bp (base pair) were prepared using the NEBNext Ultra II Directional RNA Library Prep Kit using a polyA workflow and sequenced using Novaseq 6000 (Illumina). A total of 6.5 billion reads were sequenced, with an average of 97.4 million reads per sample.

### Quality control, read mapping, and gene abundance quantification

Data preparation of the raw reads (quality control, mapping, and quantification) was carried out using the Narval cluster of the national server Compute Canada. Raw reads quality was evaluated using FastQC (v0.11.9) (Andrews 2010) and MultiQC (v1.12) (Ewels *et al*. 2016). For all samples, Phred scores were higher than 35, indicating a high quality, and thus, all reads for all samples were kept for further analysis. The duplication rate of sequences was of 64% on average, a rate considered as not unexpected in RNA-Seq libraries (Andrews 2010). Salmon mapped-based mode (Salmon/1.7.0, Patro *et al*. 2017) was run to obtain transcript level quantifications using the genome and transcriptome of a sister species, the Lake trout available from Genbank (SaNama v. 1.0; NCBI Refseq: GCF_016432855.1) (Smith *et al*. 2022). An average mapping rate of approximately 54.14% (±0.02% SD) was obtained. To use the quantification results obtained from Salmon in downstream analysis for gene-level differential expression, we used the Tximport package (v1.22.0) (Soneson *et al*. 2016) to import salmon transcript level quantifications into R studio and aggregate them to the gene level.

### Differential gene expression analysis

The differential gene expression analysis was performed using DESeq2 (v1.34) (Love *et al.* 2014). Low-expressed genes (sum of counts <=1) have been filtered out. Filtering noninformative genes may increase sensitivity detecting DEGs (Sha *et al.* 2015). The retained count data were then normalized for the library size across all samples to correct for nonbiological variation using normTransform function implemented in DESeq2, and hierarchical clustering of the 500 most expressed genes among all the experimental treatment groups was produced using the pheatmap package (v1.0.12) (Kolde 2015) to assess the global patterns of DEGs. To compare the number of DEGs resulting from the effect of parental temperature, offspring temperature, and their interactions, we used the negative binomial model implemented in DESeq2. In this model, parental temperature, offspring temperature, and their interactions were incorporated as fixed factors. This model accounts for overdispersion commonly observed in RNA-Seq count data. Only genes with an adjusted *P*-value < 0.05 after the Benjamini–Hochberg correction (DESeq2 v1.34) were considered differentially expressed and thus retained for downstream analysis. DEGs were also tabulated for upregulated and downregulated genes based on the log2fold change (LFC). For each effect, the reference group was always the cold parental temperature–cold offspring temperature group; genes with negative LFC are downregulated, and genes with positive LFC are upregulated.

### Gene ontology annotation and enrichment analysis

The GO_enrichment pipeline (https://github.com/enormandeau/go_enrichment) was used to annotate Lake trout transcript sequences by blasting them against the SwissProt protein database and retrieving the UniProt information from the UniProt website. The CSV file obtained with the go annotation information for all the transcripts sequencing was used in the Clusterprofiler package (v4.2.2) (Yu *et al*. 2012) for the enrichment analysis of the DEGs. To do so, we conducted a Gene Set Enrichment Analysis (GSEA) which involved comparing this CSV file against a gene list ranked by LFC from DESeq2 analyses to link DEGs annotations to biological processes. GSEA is based on a permutation method (with 1,000 iterations) calculating “enrichment scores”, i.e. the degree to which each predefined biological process in the whole-genome gene ontology (GO) annotation is overrepresented in DEGs. Only biological processes that included more than 10 GO terms and that had a *P*-value < 0.05 were considered significantly enriched.

## Results

**Fig. 2.**
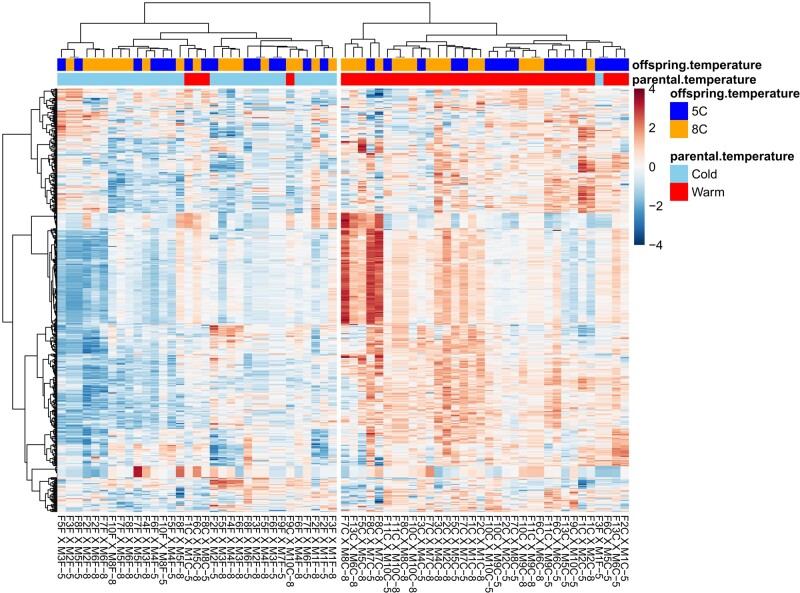
Heatmap of the 500 most expressed genes in the brain transcriptome of Brook charr fry reared either at 5 or 8 °C and whose parents were exposed to either cold or warm thermal regime during final gonad maturation stage. Each row represents a single gene, and each column represents one sample.

Hierarchical cluster analysis of the 500 most expressed genes among the four experimental groups resulted in a clear separation of brain gene-transcriptional expression in fry based on parental temperatures during final gonad maturation. However, neither offspring-rearing temperatures nor their interaction with parental temperature had a significant impact on gene expression (Fig. 2).

**Fig. 3.**
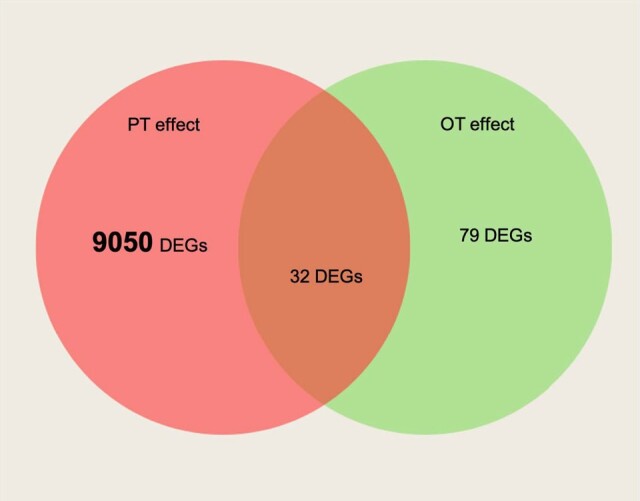
Venn diagram representing the number of differentially expressed genes (DEG) depending on parental temperature effect (PT effect), offspring temperature effect (OT effect), and the interaction effect, which is the intersection of both parental and offspring temperature cycles

**Fig. 4.**
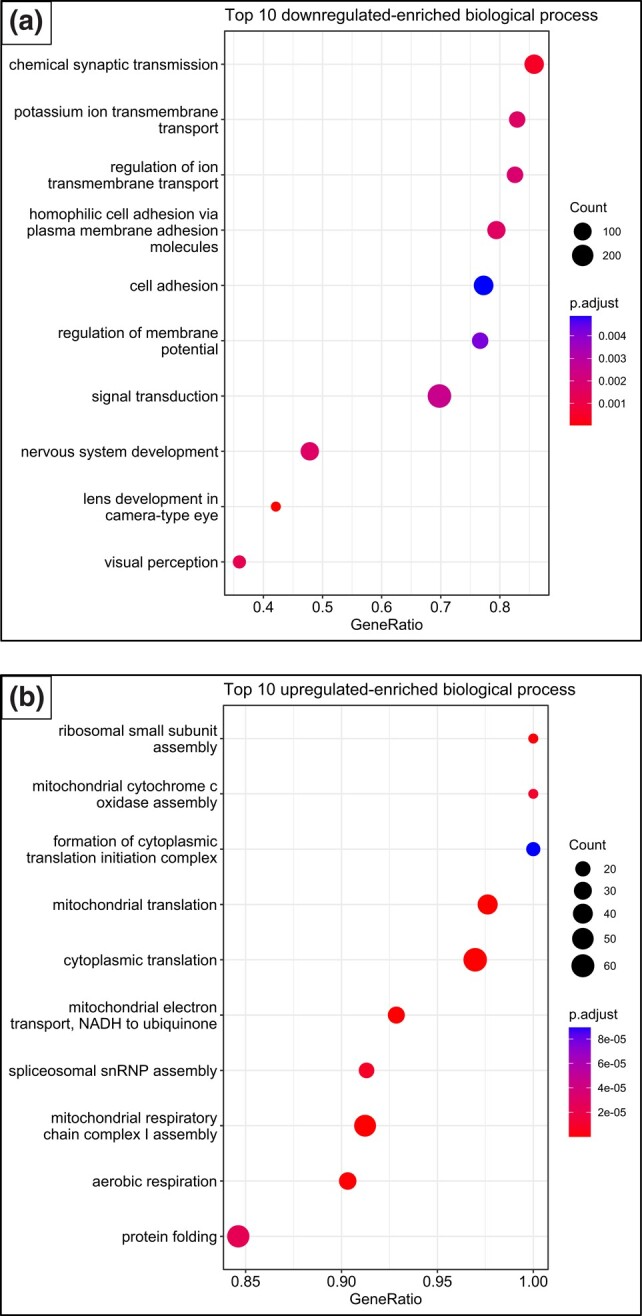
GO enrichment analysis of DEGs in the brain of Brook charr fry issued from warm-acclimated parents relative to fry issued from cold-acclimated parents (group3 vs group1); a) dot plot showing the top 10 downregulated enriched biological processes. b) dot plot showing the top 10 upregulated enriched biological processes. The size of the dots indicates the number of genes within each biological process that were found to be significantly differentially expressed. The larger the dots, the more genes are differentially expressed in the biological process. The color represents the corrected *P* value. The redder the color of the dots is, the smaller the *P*-value of the GO terms. GeneRatio refers to the proportion of genes in the genome of reference that also appear in the list of DEGs, with a higher GeneRatio indicating a greater proportion of genes involved in these processes being differentially expressed.

We identified 9,050 DEGs that mapped to 154 enriched biological processes (Fig. 3, Supplementary Table 1) when comparing fry issued from the warm parental group to those issued from the cold one (group 3 vs group1). This includes 105 functions that were significantly downregulated and 49 that were significantly upregulated. Within these DEGs, there was downregulation of processes related to neural and synaptic activity, and ion transmembrane transport; and the upregulation of some processes involved in energy metabolism, immune response, and protein folding (Fig. 4, Supplementary Tables 4 and 5).

Within the significant downregulated GO processes related to neural and synaptic activity, there were genes associated with nervous system development, chemical synaptic transmission, brain development, modulation of chemical synaptic transmission, social behavior, long-term memory, regulation of synaptic plasticity, axon guidance, and startle response (Fig. 4a, Supplementary Table 4). For the significant downregulated GO processes related to transmembrane ion activity, there were genes associated with the regulation of ion transmembrane transport, potassium ion transmembrane transport, anion transmembrane transport, and calcium ion import across the plasma membrane (Fig. 4a, Supplementary Table 4). Upregulation of energy metabolism included genes related to mitochondrial respiratory chain complex I assembly, oxidative phosphorylation, mitochondrial respirasome assembly, mitochondrial electron transport, and aerobic respiration (Fig. 4b, Supplementary Table 5). Moreover, translation, spliceosomal snRNP, and key genes involved in DNA methylation (Dnmt3a and Dnmt3b) were also significantly upregulated in fry issued from warm-parents when compared to those issued from cold parents.

Offspring-rearing temperature effect was associated with a very low number of DEGs (79 DEGs; Supplementary Table 6). No biological processes were found to be enriched within these DEGs (Fig. 3, Supplementary Table 2).

The interaction effect between parental temperature and offspring temperature resulted in only 32 DEGs that were mapped to only one downregulated biological process (visual perception) (Fig. 3, Supplementary Table 3).

## Discussion

The main objective of this study was to investigate the potential impact of elevated temperature on the whole brain transcriptome of Brook charr fry, specifically in relation to the within- and intergenerational responses to temperature exposure. As we hypothesized, parental but not offspring temperature largely affected offspring brain gene expression, corroborating DNA methylation results obtained in liver of Brook charr fry issued from the same experiment (Venney *et al*. 2022), thus confirming the intergenerational inherited epigenetic effects. As expected, neuroplasticity and processes related to adaptive thermal tolerance such as those involved in energy production, immunity, and cellular stress response were the main categories of functions affected by parental temperature increase.

### Parental temperature effect on fry brain transcriptome

The brain transcriptome of Brook charr fry was strongly influenced by parental temperature. In particular, when comparing fry that were reared at the same temperature but had parents that were exposed to different ones (warm vs cold) (group 3 vs group 1), there were approximately 116 times more DEGs than when comparing fry that were reared at different temperatures but had parents acclimated to the same thermal regime during the final stage of their sexual maturation (group 2 vs group 1). Our results thus highlight the pronounced impact of parental thermal regime in influencing offspring gene expression, irrespective of the offspring rearing temperature. Venney *et al.* (2022) found around 15 times more differentially methylated regions (DMRs) when comparing fry issued from both warm and cold parental groups compared to fry reared in different temperature conditions (464 vs 34 DMRs). Interestingly, in the brain of fry issued from the warm-parents group compared to those issued from the cold-parents group (group 3 vs group 1), we observed upregulation of Dnmt3a and Dnmt3b which encode for DNA methylation enzymes responsible for adding methyl groups (CH_3_) to DNA (Okano *et al.* 1999). To our knowledge, such an association between methylome and transcriptome when studying the effects of parental environment on progeny has not been documented in fish prior to this study, even though different tissues were compared. However, relationships between methylome and transcriptome responses were previously observed in the gill tissue of Pacific oyster (*Crassostrea gigas*) (Li *et al*. 2018; Wang *et al*. 2021). These authors found that offspring from parents living in different tidal zones exhibited distinct DNA methylation and distinct gene expression patterns in response to thermal stress, suggesting inherited epigenetic effects.

While the brain transcriptome of Brook charr fry revealed a strong influence of parental temperature, with more DEGs linked to parental thermal conditions than to the offspring temperature, this trend is not consistently observed across salmonids. For example, in Lake trout (breeders acclimated to either 10 or 17 °C from June to September; offspring exposed to either 11 or 15 °C for 3–4 weeks), Penney (2024) found a lower impact of parental temperature (202 DEGs) compared to offspring rearing temperature (365 DEGs) when examining fry liver transcriptome. In the present study, exposure of breeders to different thermal environments during final stages of gametogenesis may have contributed to an even stronger parental effect. Indeed, intergenerational epigenetic effects can occur when parents are exposed to different environments during early development or close to/during the reproduction period, due to the various epigenetic mechanisms that operate during these critical periods (e.g. Donelson *et al*. 2018). Recently, Liu *et al.* (2020) have shown that genes associated with epigenetic modifications are expressed only a few weeks before spawning in Rainbow trout (*Oncorhynchus mykiss*).

The functional annotation analysis showed an upregulation of genes involved in metabolism in offspring issued from warm parental group (group 3 vs group 1), specifically those associated with oxidative phosphorylation, aerobic respiration, and mitochondria activity, which may indicate an effect of parental thermal environments on increasing offspring mitochondria efficiency to support energy production (ATP) through oxidative phosphorylation that enables organisms’ tissues (e.g. brain) to meet the increased energy demands imposed by the warmer conditions, leading to an adaptive compensation to warming (Kotrschal *et al.* 1998). Upregulation of metabolism-related genes in offspring issued from warm-acclimated parents has been previously reported in different species such as Lake trout (Penney 2024), three-spined stickleback (*Gasterosteus aculeatus*) (Shama *et al*. 2016), and Spiny chromis (Veilleux *et al*. 2015; Bernal *et al*. 2018). At the brain level, our results indicate more enriched metabolism-related categories than what was previously observed in either liver or muscle tissues (Veilleux *et al.* 2015; Bernal *et al*. 2018; Penney 2024). The brain being a metabolically costly organ (Kotrschal *et al*. 1998) may explain these results.

In addition to metabolic responses, immune defense-related genes were significantly upregulated during intergenerational thermal acclimation in Brook charr fry. In several fish species, warm acclimation was typically linked to an increase in the expression of immune defense-related genes, such as in Rainbow trout (Rebl *et al*. 2013), Pacific salmon (*Oncorhynchus gorbuscha and Oncorhynchus nerka*) (Jeffries *et al*. 2014), Chinook salmon (*Oncorhynchus tshawytscha*) (Tomalty *et al*. 2015), and Lake trout (Penney 2024). In the current study, we found up to 60 DEGs involved in immune response in fry issued from warm-parental treatment (group 3), which could enhance their thermal stress tolerance by providing better protection against potential pathogens that may arise in warmer waters (Combe *et al.* 2023). Chaperone-mediated protein folding genes, such as those coding for heat shock proteins, were among the top 10 upregulated processes in fry issued from warm-acclimated parents. These genes are crucial biomarkers of the cellular stress response in aquatic ectotherms (e.g. Roberts *et al.* 2010), and their upregulation has been well documented in salmonids exposed to temperature increases such as Brook charr (Stitt *et al*. 2014; Mackey *et al.* 2021), Lake trout (Penney 2024), and Atlantic salmon (*Salmo salar*) (Beemelmanns *et al*. 2021). Overall, the expression of these genes could play a beneficial role in Brook charr fry, as it would prevent protein denaturation and aggregation at elevated temperatures (Fink 1999). Together, the upregulation of the most salient processes associated with thermal stress tolerance, such as metabolism, immune defense, and protein folding in fry issued from the warm parental group may support the idea of an adaptive plastic response whereby Brook charr parents may enhance the fitness of their offspring to cope with the impact of temperature increase. However, it is noteworthy that mRNA abundance may not be directly related to protein abundance and, therefore, to the phenotype observed because of the differences in protein turnover and post-transcriptional and post-translational activities (Maier *et al*. 2009).

While thermal stress tolerance-related genes were activated in fry issued from warm-acclimated parents (group 3 vs group 1), a high number of processes related to neural and synaptic activity were downregulated. This could indicate impairment of nervous system development and thereby impacts on several aspects of behavior, such as cognition, learning, memory, social interactions, and many others (e.g. O’Donnell 2018). In Rainbow trout, key genes involved in brain development, neural/cerebral disorders, and cognitive abilities were downregulated in fry originating from females exposed to elevated temperature during the preovulation period. This decrease in gene expression was associated with behavioral disorders such as inhibition of emotional reactivity and a decrease in spatial learning abilities (Colson *et al.* 2019). Nonetheless, Colson *et al*. (2019) observed a much lower number of genes exhibiting differential expression related to these activities (16 genes) than what we observed (∼4,000 genes). However, they analyzed the transcriptomic response of the entire fry, while we specifically targeted the brain. Neural and synaptic activity are some of the most energy-consuming processes, and this energy is primarily allocated toward maintaining ionic gradients across cell membranes to enable neurotransmission (e.g. Faria-Pereira and Morais 2022). As such, enough energy production must be maintained to ensure that the brain has enough energy to support neural and synaptic activity. The downregulation of such energy-demanding processes observed in our results may indicate that the upregulation of energetic metabolism is not enough to support the high energetic demands of neural activity. In line with this hypothesis, it is important to note that the variation in the genes implicated in energy production showed generally low LFC, indicating a 5–10% variation in transcription. This finding would suggest that even though Brook charr fry issued from warm-acclimated parents may have some level of thermal stress tolerance, their neural and synaptic activities may be affected, which could have long-term implications for their survival and adaptation to warmer conditions.

### Offspring rearing temperature effect on fry brain transcriptome

Offspring rearing temperature resulted in very few differences in gene expression in Brook charr fry compared to the parental effect despite previous studies in fish showing that developmental temperature may have a strong effect on gene expression including Atlantic salmon (Burgerhout *et al*. 2017), Spiny chromis (Veilleux *et al*. 2015; Bernal, Ravasi, *et al.* 2022), and in the European sea bass (*Dicentrarchus labrax*) (Anastasiadi *et al.* 2017). A certain level of environmental temperature change may be necessary to elicit a response in an organism, which is known as the threshold thermal effect (Nathan 2012). It is possible that the 3 °C difference between warm- and cold-rearing thermal regimes was not enough to elicit a significant change in gene expression in the Brook charr fry. However, we postulate that this is unlikely, as a difference of only 2 °C in the parental thermal regimes was sufficient to induce a large change in the brain transcriptome, and that early developmental life stages are known to be more thermally sensitive to environmental thermal changes than adults (Başçinar and Okumuş 2004). Hence, the lack of intragenerational plasticity observed in the current study may be attributed to the fact that the within-generational plasticity in our experiment cannot override intergenerational plasticity, suggesting a possible persistent parental effect.

### The interaction effect of parental temperature and offspring temperature on fry brain transcriptome

The interaction between parental temperature and offspring temperature resulted in only a very small effect on the gene expression pattern in the brain of Brook charr fry. This contrasts with previous observations in Spiny chromis (Veilleux *et al*. 2015; Bernal *et al*. 2018; Bernal, Ravasi, *et al*. 2022; Bernal, Schmidt, *et al*. 2022) and Lake trout (Penney 2024), which suggested that an interactive plastic response was more likely to occur when the thermal environments of the parents and offspring match which is known as the “Environmental-Matching Hypothesis” (Sheriff and Love 2013). Here, the thermal regimes experienced by parents differed by 2 °C, while the ones of offspring differed by 3 °C which is very close. We did not observe interactive effects, but the plastic response was present with the visual perception process being significantly enriched in fry reared at 8 °C and issued from warm parents (group 4 vs group 1). This enrichment could offer significant survival benefits by improving predator and/or prey detection (Howe *et al*. 2018).

## Conclusion

We investigated for the first time in a cold-adapted species the within and intergenerational responses to future temperature increase at the level of the whole brain transcriptome. Our transcriptomic results provide valuable new insights into the general response to environmental thermal stress. They corroborate results previously obtained on the methylome of offspring issued from the same crosses. Even though both were obtained on different tissues, they both highlight the predominant effect of parental temperature during final gonadal maturation on offspring compared to the offspring rearing temperature, which had only a small effect. Together, these results confirm the presence of intergenerational epigenetic inheritance.

Exposure of parents to warmer environments was associated with an upregulation of the most salient processes related to thermal stress compensation and a downregulation of several functions related to the neuronal and synaptic activity, suggesting that even though Brook charr may have some ability to adapt to warmer conditions, their behavior may still be affected. Our findings also have implications for both the aquaculture and conservation of Brook charr. By manipulating parental thermal environments, this could induce beneficial gene expression in offspring that would enhance their resilience to rising temperatures. This could improve productivity in aquaculture and increase survival rates in warmer environments. However, further research on the persistence of intergenerational effects on gene expression and downstream behavioral effects in later developmental stages of the offspring would help to better understand the potential long-term consequences for the adaptation and evolution of species in changing environments.

## Ethics

Animal husbandry, reproduction, and fry rearing were done according to Canadian Council of Animal Protection recommendations, and protocols have been approved by the UQAR Animal Care Committee (CPA-76–19-205) for parents and by the Laval University Animal Care Committee (VRR-18-111) for offspring.

## Supplementary Material

jkae051_Supplementary_Data

## Data Availability

Raw data used in this study is available at https://doi.org/10.20383/103.0894. All the scripts used in this study are available at https://figshare.com/s/9b41dade660ea778eead. Supplementary files were submitted with this manuscript. They include the global Principal Component Analysis results of gene counts depending on parental temperature, offspring, and experimental groups (Supplementary Fig. 1), the list of DEGs depending on parental temperature (Supplementary Table 1), the list of DEGs depending on offspring-rearing temperature (Supplementary Table 2), the list of DEGs depending on the interaction effect (Supplementary Table 3), the complete list of the downregulated enriched biological processes in fry issued from warm parents versus those issued from cold parents (Supplementary Table 4), the complete list of the upregulated enriched biological processes in fry issued from warm parents versus those issued from cold parents (Supplementary Table 5), and the annotation to Go terms of the 79 DEGs depending on offspring-rearing temperatures (Supplementary Table 6). Supplemental material available at G3 online.
