# Supplementary figures and images for: Parental thermal environment controls the offspring phenotype in Brook charr (Salvelinus fontinalis): insights from a transcriptomic study
Source: G3 (Bethesda). 2024 Mar 13;14(5):jkae051. doi: 10.1093/g3journal/jkae051 (PMC11075542; doi:10.1093/g3journal/jkae051)

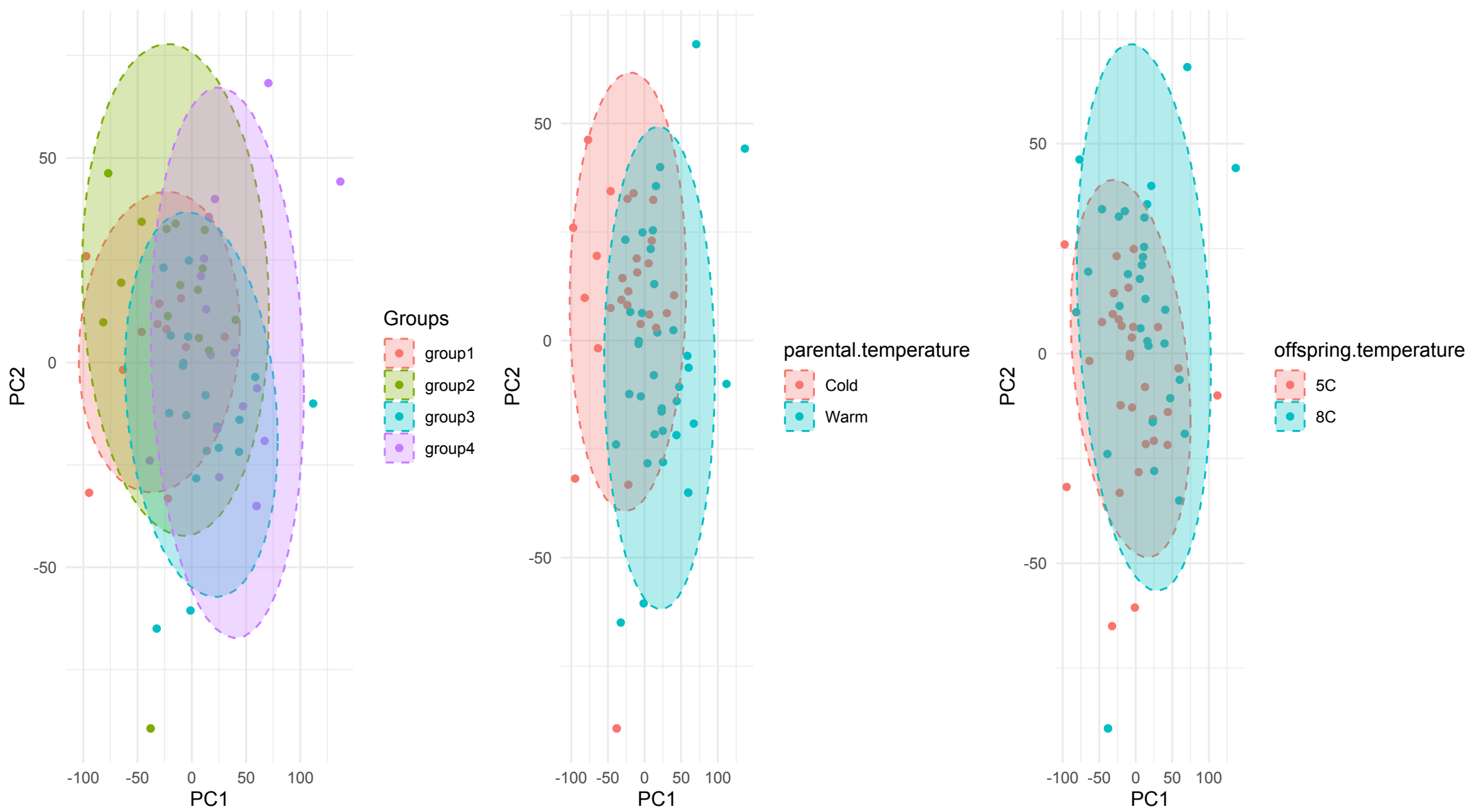

Supplement: jkae051_Supplementary_Data [file jkae051_supplementary_data.zip › Figure_S1_G3-2023-404778.pdf]
